# Joint FAO/IAEA coordinated research project on “comparing rearing efficiency and competitiveness of sterile male strains produced by genetic, transgenic or symbiont-based technologies”

**DOI:** 10.1186/s12863-020-00931-6

**Published:** 2020-12-18

**Authors:** Kostas Bourtzis, Carlos Cáceres, Marc F. Schetelig

**Affiliations:** 1 Insect Pest Control Laboratory, Joint FAO/IAEA Programme of Nuclear Techniques in Food and Agriculture, Vienna, Austria; 2 Justus-Liebig-University Giessen, Institute for Insect Biotechnology, Insect Biotechnology in Plant Protection, Winchesterstr. 2, 35394 Giessen, Germany

**Keywords:** Sterile insect technique, Insect pests, Insect disease vectors, Genetic sexing strains

Insects represent the most abundant and speciose group of animals on earth, with recent studies estimating the total number of species between 3 and 30 × 10^6^ [1, 2]. However, a small number of them represent pests for agriculture and livestock or vector major pathogens to humans and animals [3]. Area-wide integrated pest management (AW-IPM) programs with a sterile insect technique component (SIT) have been used to control populations for insect pests and disease vectors worldwide [4]. The SIT is based on the mass rearing and sterilization of insects by ionizing radiation and their subsequent handling, transport, and repeated releases, at appropriate overflooding ratios and in an area-wide manner, to suppress or locally eradicate a target population [3, 5]. Although SIT has been successfully applied by releasing both sterile males and sterile females, several studies have shown that sterile male-only releases can significantly improve the efficacy, cost-effectiveness and, in the case of mosquito SIT, also the biosecurity of SIT applications [6–8]. Male-only production can be efficiently and robustly achieved by developing specific sexing mechanisms that allow the separation of males and females in early stage prior to the release of sterile insects. The most successful example of genetic sexing strains (GSS), the VIENNA 7 and VIENNA 8 strains of the Mediterranean fruit fly, *Ceratitis capitata,* are in use worldwide for the implementation of SIT programs to control this pest [9, 10].

Sterile males can be produced by combining classical genetics and ionizing radiation, but they can also be produced by transgenic or symbiont-based approaches. The coordinated research project (CRP) entitled “Comparing rearing efficiency and competitiveness of sterile male strains produced by genetic, transgenic or symbiont-based technologies” of the Insect Pest Control Subprogramme of the Joint Division of Nuclear Techniques in Food and Agriculture of the Food and Agriculture Organization (FAO) and the International Atomic Energy Agency (IAEA) aimed: (a) to develop novel GSS or the refinement of existing ones, which may have produced by different technological platforms; (b) to perform quality control analysis of different strains mainly in respect to their rearing efficiency and mating competitiveness and (c) to assess the genetic stability of the strains by different technological platforms. This 5-year project (2015–2019) was coordinated by Kostas Bourtzis, a member of the Insect Pest Control Subprogramme of the FAO/IAEA, and included 18 scientists from 14 countries. This special issue presents the results and achievements of the research work carried out in the frame of this project.

Meza and colleagues report the development and characterization of the first GSS of the South American fruit fly *Anastrepha fraterculus sp. 1* [11]. The GSS was constructed by combining classical genetics and irradiation approaches and using the *black pupae* (*bp*) gene as a selectable marker. In the new strain, males emerge from brown pupae and females from black pupae. This GSS sets the ground for the development and implementation of SIT applications against this major agricultural pest in South America. Aketarawong and colleagues evaluated the Salaya 1 GSS of the Oriental fruit fly *Bactrocera dorsalis* based on the *white pupae* (*wp*) gene selectable marker, under semi-mass rearing conditions [12]. The results clearly showed that the genetic stability, genetic diversity, and the overall biological quality of this GSS, which was also developed by the combination of classical genetics and irradiation approaches, is maintained during the production in the mass rearing facility by using protocols to rearing GSS strains included the set-up of the filter rearing system that ensures the integrity of the sexing characteristics of the strain.

The Queensland fruit fly, *Bactrocera tryoni*, represents a major agricultural pest for Australia. There are ongoing efforts to develop and implement a large-scale operational SIT project for its population control. During this CRP, Choo and colleagues used CRISPR/Cas9-based approaches to introduce single point mutations in the *shibire* gene to use it as a conditional selectable marker for the development of GSS for this pest species [13]. Popa-Baez and colleagues applied the “common garden” approach *in B. tryoni* to investigate naturally occurring genetic variation in traits such as heat, desiccation, and starvation resistance and to assess the impact of domestication on them [14]. Their results clearly show that variation exists, and it is possible to identify populations with key traits for SIT applications, such as high heat, desiccation, and starvation resistance. However, the variation declines very fast during domestication, indicating the need for careful selection plans to minimize the loss of useful traits. Yeap and colleagues studied two defining characters, mating time and callus color, in the sibling species *Bactrocera tryoni* and *Bactrocera neohumeralis* using hybrid recombinant analysis [15]. Their results indicated that the two traits, although tightly linked, can be separated, and each respective phenotype is determined by more than one gene. From an applied point of view (domestication, mass rearing, and SIT applications), it is important to note that the *Bactrocera tryoni* phenotypes are selected under laboratory conditions. Further on, research efforts were also focused on another *Bactrocera* species, the olive fruit fly *Bactrocera oleae*. Tsoumani and colleagues report on the identification and characterization of the *odorant receptor co-receptor* (*orco*) gene, which encodes for the Orco [16]. This olfactory co-receptor plays a crucial role in the mating behavior of the olive fruit fly, at both pre- and post-mating level, and represents a target for the development of novel control strategies in support of SIT. Carraretto and colleagues report on the discovery of repetitive sequences on the Y chromosome of *Bactrocera dorsalis* which may set the ground for a useful marking system and molecular karyotyping in this species [17].

The spotted wing *Drosophila*, *Drosophila suzukii*, is an invasive species and a major agricultural pest, and has been the target of development for novel genetic control strategies. In the present special issue, Yan and colleagues present the isolation and characterization of four cellularization genes *nullo, serendipity-α, bottleneck,* and *slow-as-molasses* including their promoters [18]. Given that these genes are active during embryogenesis, their promoters can be used to express factors for the induction of sterility or for sexing in support of SIT applications against this pest species. In addition, Ahmed and colleagues showed that higher *piggyBac* germline transformation efficiency can be achieved by using the *D. suzukii* Alps line compared to others [19]. In the same study, the authors also constructed several φC31-RMCE, early embryo-specific, and spermatogenesis-specific driver lines, which can be proven useful for future genetic engineering efforts for the population control of this pest.

Novel tools were also developed for other insect species. Primo and colleagues performed Cas9-sgRNA injections into *Ceratitis capitata* XX-only embryos targeting the autosomal transformer (*tra*) gene [20]. This resulted in the production of XX males as expected. However, the authors noted that some of the XX males did not have any mutations in the *tra* gene clearly indicating that the Cas9-sgRNA injections or the injection of inactive Cas9 can lead to masculinization without the induction of mutations. Handler and Schetelig used the transposable *hopper* element isolated from *Bactrocera dorsalis* to successfully transform *Drosophila melanogaster* and the SIT target pest species the Caribbean fruit fly, *Anastrepha suspensa* [21]. Scannapieco and colleagues report on the transcriptomic analysis of embryos, males, and females of *Anastrepha fraterculus sp. 1,* thus providing useful information for the development, courtship, and reproduction of this species [22]. This study together with the one by Giardini and colleagues, which reports on the cytogenetic analysis with an emphasis on sex chromosomes polymorphism, provides useful tools for the development of genetic control methods against the South American fruit fly *Anastrepha fraterculus sp. 1* [23].

In the frame of the CRP, research studies on fruit fly symbionts were also carried out. Cai and colleagues present a comparative genomic analysis of the gut-associated symbiont of *Bactrocera dorsalis*, a member of the *Klebsiella* genus, which sheds light on host-symbiont interactions and the role this symbiotic bacterium may play in the biology and physiology of the Oriental fruit fly [24]. Nikolouli and colleagues studied the gut-associated bacterial species and the genetic profile of natural *C. capitata* populations originating from Europe, Africa, Australia, and the Americas [25]. The results showed clear genetic differentiation while reduced diversity was detected in the associated microbiota indicating the important role of geographic expansion and colonization pathways.

Significant progress was achieved during this CRP with respect to the development of male-only strains for two major livestock pests: *Lucilia cuprina*, a major pest of sheep, and *Cochliomyia hominivorax*, a major pest of warm-blooded animals. The first study by Yan and Scott reports on the construction and characterization of a Tet-off based transgenic sexing strain of *Lucilia cuprina*, which exhibits enhanced stability and reduced risk for resistance because the authors combined two lethal effectors in a single strain [26], Concha and colleagues present the construction and characterization of several transgenic sexing strains for the New World screwworm, *Cochliomyia hominivorax*, using an early female lethal system, which could potentially enhance the SIT applications against this major pest [27].

An interesting recent expansion has been the development of genetic sexing strains for a major mosquito vector species, *Aedes aegypti*, using classical genetic approaches [28]. A key parameter in GSS is genetic stability, which can be affected by recombination events. In this issue, Augustinos and colleagues used ionizing radiation to induce chromosomal inversions, known as suppressors of genetic recombination [29]. One of them (Inv35) was subsequently used for the construction of a red-eye GSS in *Aedes aegypti*, which can be used in SIT applications for the population suppression of this vector to fight against major pathogens such as dengue, chikungunya, Zika, and yellow fever.

As guest editors, we believe that this issue of BMC Genetics presents significant information on how GSS can be developed, refined, and evaluated for their biological quality and potential use in large-scale operational population control programs against insect species and disease vectors. In addition, recent discoveries of the *Maleness-on-the-Y* (*MoY*) gene, the male determining factor in Tephritids, and the *white pupae* (*wp*) gene, which has been used as a selectable marker in several tephritid GSS, set the ground for the development of generic approaches for the construction of GSS against insect pests and disease vectors [30, 31]. This will be the focus of a new 5-year long (2019–2024) Coordinated Research Project on “Generic approach for the development of genetic sexing strains for SIT applications”.

## Abbreviations

AW-IPM: Area-wide integrated pest management
CRP: Coordinated research project
FAO: Food and Agriculture Organization of the United Nations
GSS: Genetic sexing strains
IAEA: International Atomic Energy Agency
SIT: Sterile insect technique component
